# Circadian Genes as Exploratory Biomarkers in DMD: Results From Both the *mdx* Mouse Model and Patients

**DOI:** 10.3389/fphys.2021.678974

**Published:** 2021-07-08

**Authors:** Rachele Rossi, Maria Sofia Falzarano, Hana Osman, Annarita Armaroli, Chiara Scotton, Paola Mantuano, Brigida Boccanegra, Ornella Cappellari, Elena Schwartz, Anton Yuryev, Eugenio Mercuri, Enrico Bertini, Adele D’Amico, Marina Mora, Camilla Johansson, Cristina Al-Khalili Szigyarto, Annamaria De Luca, Alessandra Ferlini

**Affiliations:** ^1^Unit of Medical Genetics, Department of Medical Sciences, University of Ferrara, Ferrara, Italy; ^2^The Dubowitz Neuromuscular Centre, Institute of Child Health, London, United Kingdom; ^3^Department of Medical Microbiology, Faculty of Medical Laboratory Sciences, University of Khartoum, Khartoum, Sudan; ^4^Section of Pharmacology, Department of Pharmacy-Drug Sciences, University of Bari “Aldo Moro”, Bari, Italy; ^5^Ami-Go-Science LLC, Rockville, MD, United States; ^6^Elsevier, Rockville, MD, United States; ^7^Pediatric Neurology Unit, Catholic University and Nemo Center, Policlinico Universitario Gemelli, Rome, Italy; ^8^Unit of Neuromuscular and Neurodegenerative Disorders, Department of Neurosciences, IRCCS Bambino Gesu Children’s Hospital, Rome, Italy; ^9^Neuromuscular Diseases and Neuroimmunology Unit, Fondazione IRCCS Istituto Neurologico Carlo Besta, Milan, Italy; ^10^School of Chemistry, Biotechnology and Health, Royal Institute of Technology, Stockholm, Sweden; ^11^Science for Life Laboratory, Royal Institute of Technology, Stockholm, Sweden

**Keywords:** circadian rhythm, Duchenne muscular dystrophy (DMD), *mdx* mice, skeletal muscle, RNA analysis, biomarker

## Abstract

Duchenne muscular dystrophy (DMD) is a rare genetic disease due to dystrophin gene mutations which cause progressive weakness and muscle wasting. Circadian rhythm coordinates biological processes with the 24-h cycle and it plays a key role in maintaining muscle functions, both in animal models and in humans. We explored expression profiles of circadian circuit master genes both in Duchenne muscular dystrophy skeletal muscle and in its animal model, the mdx mouse. We designed a customized, mouse-specific Fluidic-Card-TaqMan-based assay (Fluid-CIRC) containing thirty-two genes related to circadian rhythm and muscle regeneration and analyzed gastrocnemius and tibialis anterior muscles from both unexercised and exercised *mdx* mice. Based on this first analysis, we prioritized the 7 most deregulated genes in mdx mice and tested their expression in skeletal muscle biopsies from 10 Duchenne patients. We found that *CSNK1E, SIRT1*, and *MYOG* are upregulated in DMD patient biopsies, consistent with the mdx data. We also demonstrated that their proteins are detectable and measurable in the DMD patients’ plasma. We suggest that *CSNK1E, SIRT1*, and *MYOG* might represent exploratory circadian biomarkers in DMD.

## Introduction

The dystrophin gene (DMD) (OMIM 300377), located in Xp21.2-p21.1, is a 2,2 Mb gene that encodes for the dystrophin protein (DYS), a subsarcolemmal, rod-shaped protein of 427kDa involved in the formation of the dystrophin-associated protein complex (DAPC) (Ervasti and Campbell, 1993). The DAPC is composed of dystroglycans, sarcoglycans, sarcospan, dystrobrevins, and syntrophin. This complex exerts the structural function of mechanic-transducer between muscle fibers and the extracellular matrix and controls membrane stability. The multiple binding sites and domains present in the DAPC confer the scaffold of various signaling and channel proteins, which may implicate the DAPC in regulation of signaling (Constantin, 2014).

A large variety of dystrophin gene mutations (approximately 75% of deletions/duplications and 25% of small/atypical mutations) cause dystrophinopathies (see in DMD Leiden pages^1^). Mutations maintaining the reading frame are generally associated with Becker muscular dystrophy (BMD) and other milder or atypical allelic forms of dystrophinopathies (OMIM 300376). In contrast, frameshifting mutations are mainly associated with severe Duchenne muscular dystrophy (DMD OMIM 310200) and cause a complete absence or severe reduction of the dystrophin protein. Some exceptions to the rule occur via diverse mechanisms such as splicing modulation or novel ATG start site use (Gualandi et al., 2006; Ferlini et al., 2013; Wein et al., 2015). Dystrophin-deficient fibers are more prone to membrane damage following muscle contraction. The resulting leaky membrane leads to the onset of a calcium-mediated degenerative process that culminates in inflammation, oxidative stress pathway activation, and consequent muscle fiber necrosis (De Luca et al., 2002). At the early stage, the regenerative process (myoblast recruitment and differentiation) is able to counterbalance the damage. However, after a number of degenerative-regenerative cycles, regeneration fails and there is the complete fibrotic substitution of muscle tissue (Massopust et al., 2020).

Duchenne muscular dystrophy is the most severe form of dystrophinopathies and is clinically characterized by muscle wasting with onset in early childhood (around 3-4 years old) and a progressive course that culminates in death by cardiac or respiratory complications around 20-30 years old (Merlini and Sabatelli, 2015).

In recent years, novel therapeutic approaches have emerged that aim at ameliorating the disease’s course, even if an etiologic cure has not been discovered to date (Sun et al., 2020). Steroids (prednisolone and deflazacort) remain, however, the gold-standard drugs.

These new ongoing clinical trials in DMD would greatly benefit from the use of specific biomarkers (Scotton et al., 2014) since reliable biomarkers would facilitate not only correct disease diagnosis, but also disease monitoring and prognosis, patient stratification, and the prevision of an individual drug response. Furthermore, sensitive and feasible biomarkers could improve drug screening and effectiveness evaluations.

The most used animal model of dystrophinopathies is the *mdx* mouse that carries a point mutation in exon 23, leading to a stop codon instead of a glutamine. Despite the absence of dystrophin, the overall phenotype of this mouse model is less severe with respect to Duchenne muscular dystrophy (Lynch et al., 2001).

Evidence highlights the role of exercise in modifying skeletal muscle pathology in *mdx* mice, showing a different impact in different muscle types, that contributes to a worsening of the overall muscle phenotype, prolong the degenerative phase, and enhance DMD-like muscle pathology (Grounds and Torrisi, 2004; Camerino et al., 2014; Capogrosso et al., 2016).

We have identified a relationship between muscle damage and disease severity and circadian genes, suggesting that the *CLOCK* gene represents a disease severity biomarker in collagen VI myopathies (Scotton et al., 2016).

Here, we have investigated circadian gene behavior in Duchenne muscular dystrophy (DMD) and its animal model, the mdx mouse. Both exercised and untrained mdx mice and the skeletal muscle of DMD patients were studied by RNA profiling and a pilot immunoassay study was carried out on selected protein in 10 DMD boys.

We found a general deregulation of circadian key genes in mdx muscles. Based on mice results, we prioritized 7 genes and demonstrated that *CSNK1E*, *SIRT1*, and *MYOG* are invariably upregulated in DMD skeletal muscle and also measurable in plasma. We suggest that these circadian genes may represent exploratory biomarkers for DMD and might underline an interesting link between DMD pathology and circadian rhythm.

## Materials and Methods

### Animal Selection and RNA Extraction

*In vivo* experiments and animal housing were carried out in conformity with the Italian Guidelines for Care and Use of Laboratory Animals (D.L. 116/92) and with the European Directive (2010/63/UE). The study was approved by the National Ethics Committee for Research Animal Welfare of the Italian Ministry of Health (authorization no. 1119/2020-PR). Experimental procedures were conducted according to standard operating procedures for pre-clinical tests in *mdx* mice, the SOP (ID) Number DMD_M.2.1.001, available at the TREAT NMD website https://treat-nmd.org/wp-content/uploads/2016/08/MDX-DMD_M.2.1.001.pdf.

In this study, a total of 6 male wild type (WT; C57BL/10ScSnJ) and 13 male *mdx* (C57BL/10ScSnDmd<mdx>/J) mice aged 4-5 weeks (Charles River, Italy for Jackson Laboratories) and homogeneous for their body weight, were selected. The *mdx* mice were divided into two groups: the sedentary group consisting of 6 male *mdx* mice and the exercised group consisting of 7 male *mdx* mice. The exercise protocol was performed as previously described (Camerino et al., 2014) by running for 30 min on a horizontal treadmill (Columbus Instruments) at 12 m/min, twice a week (keeping a constant interval of 2–3 days between each trial). Both *wt* and sedentary *mdx* mice were left to move freely in the cage without any exercise. At 8-9 weeks of age, gastrocnemius (GC) muscle was collected from a total of 10 mice, 3 wt and 7 *mdx*. Of the mdx mice, 3 were sedentary and 4 exercised by treadmill exercise for 4 weeks (see Table 1).

**TABLE 1:** Wt and *Mdx* mice cohorts.

**Mice cohorts**		**Gastrocnemius (Age 8-9 weeks)**	**Tibialis anterior (Age 16-17 weeks)**
wt		3	3
*mdx*	Sedentary	3	3
	Exercised	4	3

*A 6 wt and 13 *mdx* mice were selected. The 13 *mdx* were divided into 2 groups, sedentary and exercised; in 7 *mdx* mice, GC muscle was collected at the age of 8-9 weeks, following 4 weeks of exercise; in the other 6 *mdx* mice, TA muscle was collected at the age of 16-17 weeks, following 12 weeks of exercise.*

The fast-twitch tibialis anterior (TA) muscle was collected in the remaining 9 mice at 16 to 17-weeks-old (3 wt, 3 *mdx*, and 3 *mdx* exercised for 12 weeks, see Table 1, in the attempt to match pathology phase in the two different muscle types).

Muscles were removed from anesthetized mice (100 mg/kg ketamine and 16 mg/kg xylazine intraperitoneal) at the same time frame (8-11 a.m.), washed in PBS, and rapidly frozen in liquid nitrogen-cooled isopentane and stored at -80°C until use. Sampling time has been selected according to ethic rules and approved experimental plan.

Total RNA was isolated using the RNeasy-kit (Qiagen, Chatsworth, CA, United States) according to manufacturer’s instructions and treated twice with DNase (RNase free DNAse set Qiagen kit) to exclude possible genomic contamination. A DNA contamination check was performed using a Real-time PCR system designed on murine actin-b exon 3. Nucleic acid concentration was quantified using a Nanodrop (Thermo Fisher Scientific) spectrophotometer.

### Custom Fluidic Cards Exploring Circadian Genes (Mus Musculus Fluid-CIRC) Design and Circadian Gene Expression Analysis in *mdx* Mice

To test in mouse model the effect of DMD disease on circadian rhythm, we selected murine genes for the sub-network enrichment analysis base on the Kotelnikova work (Kotelnikova et al., 2012).

Specifically, the 32 murine genes chosen (Table 2) are involved in circadian rhythm, muscle regeneration, metabolism, apoptosis, immune reaction, and cellular proliferation.

**TABLE 2:** Selected genes for Fluid-CIRC design.

**Gene code**	**Gene name**	**Function**
Arntl1	Aryl Hydrocarbon Receptor Nuclear Transclocator-Like1	Transcription factor, CCGs component of positive loop
Arntl2	Aryl Hydrocarbon Receptor Nuclear Transclocator-Like2	Transcription factor, CCGs component of positive loop
Atf5	Activating Transcription Factor 5	Transcriptional repressor; blocks the differentiation of neuronal progenitor cells
18s rRNA	ribosomal RNA 18s	Component of 40s minor subunit of ribosome
Ccrn4l	Carbon Catabolite Repressor 4-like (Nocturnin)	Deadenylase: plays an important role in post-transcriptional regulation of metabolic genes under circadian control.
Clock	Circadian Locomotor Output Cycles Kaput	Core clock gene, involved in the positive arm of the transcriptional-translational feedback loop.
Dbp	D-Site Binding Protein	Transcriptional activator, not crucial for circadian rhythm but modulates important clock output genes
Egr1	Early Growth Response 1	Transcriptional regulator
Fkbp5	Fk506 Binding Protein 5	Regulator of trafficking of steroid receptor containing vesicles
Per1	Period circadian clock 1	CCGs component of the negative transcriptional-translational regulatory negative loop
Per2	Period circadian clock 2	CCGs component of the negative transcriptional-translational regulatory negative loop
Per3	Period circadian clock 3	CCGs component of the negative transcriptional-translational regulatory negative loop
Cry1	Cryptochrome Circadian Clock1	Transcriptional repressor of circadian positive loop. It translocates PER proteins into the nucleus
Cry2	Cryptochrome Circadian Clock1	Transcriptional repressor of circadian positive loop. It translocates PER proteins into the nucleus
Rorα	RAR-Related Orphan Receptor A	Transcriptional factor that regulates lipid metabolism, circadian rhythm, and skeletal muscle differentiation
Nr1d1	Nuclear Receptor Subfamily 1, Group D, Member 1	Transcriptional repressor of Clock, Arntl1, Cry1
Nr1d2	Nuclear Receptor Subfamily 1, Group D, Member 2	Transcriptional repressor of Clock, Arntl1, Cry1
Csnk1ε	Casein Kinase 1, Epsilon	Kinase that phosphorylates many proteins, among which circadian proteins Per1 and 2
Csnk1δ	Casein Kinase 1, Delta	Kinase that phosphorylates many proteins, among which circadian proteins Per1 and 2
Bhlhe40	Basic Helix-Loop-Helix Family, Member E40	Transcriptional Factor which interacts with Arntl and indirectly modulates Per1 transactivation via Clock/Arntl1
Bhlhe41	Basic Helix-Loop-Helix Family, Member E41	Transcriptional repressor
Tim	Timeless circadian clock	Transcriptional repressor of circadian genes involved in the positive loop
Sirt1	Sirtuin1	Deacetylase involved in many different functions such as DNA repair, metabolism, apoptosis, and autophagy
Myod1	Myogenicenic Differentiation 1	Transcriptional activator of muscle-specific genes mainly involved in muscle differentiation. It regulates myogenesis
Myog	Myogenin (Myogenic factor 4)	Transcriptional activator of many muscle-specific genes. It plays a role in end-stage muscle differentiation and adult muscle phenotype
Dmd	Dystrophin	Muscle specific structural protein
Ppargc1α	Peroxisome Proliferator Activated Receptor Gamma, Coactivator 1α	Transcriptional co-activator of steroid and nuclear receptors; has a role in fatty acid and glucose metabolism
Tgfb1	Transforming Growth factor, Beta 1	Controls cellular proliferation and differentiation
Gapdh	Glyceraldehyde-3-Phosphate dehydrogenase	Role in glycolysis, transcription, RNA transport, DNA replication, and apoptosis
ActB	Actin, Beta	Globular protein, it forms thin filaments of sarcomere

*Gene-specific functions are listed; CCGs, core clock gene components.*

We created a specific, custom TaqMan Low Density Array (TLDA) micro fluidic-card, Fluid-CIRC, combining ABI TaqMan gene assays (Applied Biosystems, Foster City, CA, United States) inventoried for the 32 selected murine gene with *GAPDH*, β*-actin* (*Actb*), and *18s* as reference genes. In the chosen design, all genes were run in triplicate and 4 samples were run in each card (Supplementary Figure 1).

For each muscle sample of all mice cohorts, a total of 300 ng of RNA were reverse-transcribed using the High-capacity cDNA Reverse Transcription Kit (Applied Biosystems) and then added to 100 μl of Real-time Universal PCR Master mix. Sterile water was added to reach a total volume of 200 μl and the final solution was loaded in 2 ports (100 μl each) of the Fluid-CIRC and run on ABI 7900HT System Fast Real-time PCR System (Applied Biosystems) using the following conditions: 2 min. at 50°C, 10 min. at 95°C, 40 cycles at 97°C for 15 s. then 1 min. at 60°C (Applied Biosystems TaqMan Array Micro Fluidic Cards user Guide).

Normalization was performed using Actn-B as housekeeping reference. For both muscles (gastrocnemious and tibialis anterior) the average of the DCt of the total number of samples was calculated for each mice cohort (wt, *mdx*, and *mdx* exercised, Table 1) and the statistical analysis was performed using Graphpad calculator^2^. For each gene the mean standard error (SEM) was calculated as shown in Supplementary Figure 2, and t-test was performed to determine statistically significant gene expression variation (*p*-Value < 0.05) (Supplementary Figure 2).

Mice were subdivided into 2 age-matched cohorts, wild type (WT) and *mdx* exercised (*mdx-exe*) for the two muscles (GC and TA), and were compared as *mdx* vs. WT and *mdx*-exe vs. *md*x.

### Gene Prioritization Analysis

Gene prioritization analysis was performed using Gene Set Enrichment Analysis based on the well-established approach of ranking genes by *p*-Values associated with the phenotype (a uniform distribution using a weighted Kolmogorov-Smirnov test as previously described in Kotelnikova et al., 2012). Using a sub-network enrichment analysis (SNEA), significantly deregulated genes based on *p*-Values and concordant in terms of side of expression change were identified and selected relative to their crucial role as downstream effectors in the core clock circadian network. Interactome was implemented in Pathway Studio.

### DMD Patient Studies

#### Expression Analysis of Prioritized Genes in DMD Patients’ Muscle

##### Patient selection, RNA extraction, and real-time PCR analysis

Ten DMD patients with different mutation types and with variable severity phenotypes were enrolled in this study. Mutations and clinical characteristics are detailed in Table 3. From each DMD subject, we obtained a muscle biopsy (tibialis anterior) either by in-house diagnostic procedures or via the Telethon biobank. In all cases, written informed consent was obtained and specific approval of this research study from the S. Anna University Hospital of Ferrara Ethics Committee (no. 02/2009, 26th February 2009) was achieved. This research was conducted following the Declaration of Helsinki’s rules concerning human subject research. As a control, we used a pool of RNA extract from 3 healthy control samples [Control 1: commercial human skeletal muscle total RNA (Ambion) male, 71 years; Control 2: healthy donor male, 9 years; Control 3: healthy donor male, 37 years].

**TABLE 3:** Duchenne muscular dystrophy patients selected for expression analysis of the 7 most deregulated genes.

**Patient**	**Mutation**	**Phenotype**	**Age at muscle biopsy sampling**
PT1	Deletion exons 46-55	DMD	13 years
PT2	Deletion exons 61-63	DMD	11 years
PT3	Duplication exons 5-7	DMD	11 years
PT4	c.2950-2A > G	DMD	7 years
PT5	c.9808-1G > A	DMD	4 years
PT6	c.3655-3656indelGG > TT, p.E1150X	DMD	4 years
PT7	c.2510C > T, p.R768X	DMD	8 years
PT8	c.158T > A c.2971G > C	DMD	4 years
PT9	c.8027 + 2T > A	DMD	4 years
PT10	c.10223 + 2T > C	DMD	4 years

*Age at muscle biopsy sampling and mutation are detailed.*

Muscle sample collecting procedures were carried out following local standard surgical procedures between 8 and 10 a.m. (CET) and frozen shortly afterward in liquid nitrogen until use. The concordance of collection time for each sample allowed us to correctly analyze and compare molecular clock component genes.

Total RNA was isolated from all muscle specimens using an RNeasy-kit (Qiagen, Chatsworth, CA) according to the manufacturer’s instructions, double-treated with DNase (RNase free DNAse set Qiagen kit) and reverse-transcribed using the HighCapacity cDNA Reverse Transcription Kit (Applied Biosystems). DNA contamination checks were performed using a Real-time PCR system designed in intron 14 of the dystrophin gene. Nucleic acid concentration was quantified using the Nanodrop (Thermo Fisher Scientific) spectrophotometer.

Transcript quantification of the 7 selected circadian genes *CNSK1E, SIRT1*, *MYOG, MYOD1, CRY1, CRY2*, and *ARNTL* was obtained using commercially available TaqMan expression assays (Applied Biosystems): CSNK1E, NM_001894.4, Hs00266431_m1, exon boundaries 6-7; SIRT1, NM_001142498.1, Hs01009006_m1, exon boundaries 7-8; MYOG, NM_002479.5, Hs01072232_m1, exon boundaries 2-3;MYOD1, NM_002478.4, Hs02330075_g1, exon boundaries 1-2; CRY1, NM_004075.4, Hs00172734_m1 exon boundaries 2-3; CRY2, NM_001127457.1, Hs00323654_m1, exon boundaries 5-6; ARNTL, NM_001030272.1, Hs00154147_m1, exon boundaries 8-9. Genes were selected according to the most deregulated genes observed in the mice Fluidic cards data.

β-actin was selected as a housekeeping reference gene (ACTB Endogenous Control). All Real-Time reactions were run in triplicate. Data were analyzed according to the comparative CT method (2^–ΔΔ*Ct*^ method). and statistical analyses were performed with students’*t*-test using the technical replicates for both control and DMD patients.

#### CSNK1E, SIRT1, and MYOG Protein Quantification in Plasma

##### CSNK1E ELISA assay

Plasma samples from 16 patients (2 BMDs and 14 DMDs) with 6 age-matching control males, were collected after written informed consent and approval by the Ethics Committee of S. Anna University Hospital of Ferrara (no. 02/2009, Feburary 26, 2009, BIO-NMD European Union Seventh Framework Programme). Genotypic and phenotypic information are summarized in Table 4.

**TABLE 4:** Selected patients for Csnk1ε protein quantification in plasma.

**Code**	**Mutation**	**DMD/BMD**	**Age at loss of ambulation**	**Age at plasma sampling**
A	del exons 3-7 (out of frame)	BMD	20 years	33 years 7 m
B	del exon 13	BMD	Ambulant at sampling	7 years 2 m
C	del exon 43	DMD	Ambulant at sampling	8 years
D	del exon 45	DMD	Ambulant at sampling	9 years
E	del exon 45	DMD	Ambulant at sampling	9 years
F	del exon 45	DMD	Ambulant at sampling	6 years
G	del exon 45	DMD	Ambulant at sampling	7 years
H	del exon 45	DMD	Ambulant at sampling	6 years
I	del exon 45-50	DMD	Ambulant at sampling	10 years
L	del exon 45-50	DMD	Ambulant at sampling	12 years
M	del exon 45-50	DMD	Ambulant at sampling	7 years
N	del exon 49-50	DMD	Ambulant at sampling	7 years
O	del 50	DMD	Ambulant at sampling	10 years
P	dup exons 65-79	DMD	Ambulant at sampling	19 years
Q	c.4117c > T, p.Q1373X	DMD	Ambulant at sampling	6 years
R	c.9204-9207del,p.N3068K, fs*20	DMD	Ambulant at sampling	8 years

*Mutation, phenotype and age at sampling are listed.*

Plasma was isolated from peripheral blood after a single centrifugation within 2 h after sampling at 1500 g for 10 min at 4°C and immediately stored at -80°C in 400 ul aliquots.

ELISA assay was performed using the CSNK1E ELISA kit (My BioSource) according to the manufacturer’s instructions. In brief, a total of 100 μl of standard and samples (in triplicate) were added to a pre-coated microplate (96 wells) with the antibody specific for CSNK1E and incubated for 2 h at 37°C. Following incubation, steps were performed with100 μl of Biotin-antibody (1×), 100 μl of HRP-avidin (1×), and 90 μl of TMB substrate. All the incubations were at 37°C, the first two for 1 h and the last one for 15 min. Finally, 50 μl of stop solution was added and the optical density of each well was determined using a microplate reader at 450 nm within 5 min.

For data analysis, a standard curve was constructed using “Curve expert 1.3” software, according to the manufacturer’s instructions. CSNK1E concentration was calculated based on the absorbance value in relation to the calculated standard curve according to the equation of [CSNK1E] = (mean absorbance - 0,0391)/0,0013 and finally expressed in pg/ml.

CSNK1E data analysis was performed using R software.

##### SIRT1 and MYOG detection by suspension bead assays

A 44 DMD, 9 BMD, and 28 control plasma samples, coming from the large BIO-NMD cohort already reported (Ayoglu et al., 2014), were analyzed to assay SIRT1 and MYOG using the suspension bead array (LUMINEX Corp.). Two antibodies toward SIRT1 (HPA006295 and HPA007016) and one toward MYOG (HPA038093) (Uhlén et al., 2015) were diluted to a 17.5 μg antibody per ml in a 0.1 M 2-(N-morpholino) ethanesulfonic acid (MES) buffer (pH 4.5). Antibodies were then coupled to carboxylated, fluorescently labeled, magnetic beads (MagPlex-C, Luminex Corp.) according to previously established protocol (Ayoglu et al., 2014) with minor changes. After the coupling of antibodies, the beads were re-suspended in a 50 μl storage buffer containing 5% w/v bovine serum albumin (Albumin fraction V from bovine serum, Merck KGaA, 1.12018.0100) 0.05% v/v Tween 20 in PBS and incubated for 2 h at room temperature. Raw plasma samples from DMD and BMD patients, as well as healthy control samples, were biotinylated according to previously described protocols (Ayoglu et al., 2014) and diluted to a final dilution of 1:708 in 50 μl of an assay buffer containing 0.52 mg/ml bovine IgG (I-5506, Sigma) and 0.01% v/v ProClin^TM^ 300 (48912-U, Sigma) in PBST. The diluted samples were incubated together with beads overnight at 4°C, followed by isolation and washing of the beads in PBST. Streptavidin R-phycoerythrin conjugate was used for detection. Analysis was performed using a Luminex 100/200 instrument (Luminex Corp.) with Luminex xPONENT software. Statistical analysis of mean fluorescence intensity (MFI) was performed using packages *ggsignif* and *ggplot2* in R (Gatto et al., 2015). Wilcoxon ranked sum test was performed to assess differences in MFI between patient groups. Assays were performed in triplicates. Reproducibility was assessed at three different concentrations.

## Results

### Circadian Genes Are Invariantly Deregulated in *mdx*

In order to explore the involvement of circadian genes in dystrophinopathies, we designed a custom TaqMan Low Density Array (TLDA) micro fluidic-card, Fluid-CIRC, to obtain the transcriptional profiling of two different *mdx* muscles: gastrocnemius (GC) and tibialis anterior (TA) derived from both sedentary and trained mice. These muscles were selected because of their involvement in *mdx* pathology and, for GC in particular, its muscle fiber composition (both fast and slow fibers are represented) and its large and early functional involvement in horizontal treadmill exercise as applied in our study (Camerino et al., 2014, TREAT-NMD^3^).

#### Sedentary *mdx* vs. Sedentary WT Mice

Gastrocnemius and TA muscles from 3 different *mdx* were compared with age-matched (8-9 weeks for GC and 16-17 weeks for TA) WT muscles (all sedentary), for the differential expression of selected circadian genes (Table 1).

As shown in Supplementary Figure 2A (sedentary *mdx* mice vs. sedentary WT mice) there is a general trend toward a downregulation of all core clock genes. In particular, a statistically significant (*p*-Value < 0.05) downregulation affects Ccrn4l, Fkbp5, Per3, Cry1, Ror-α, Nr1d1, Nr1d2, Csnk1ε, Sirt1, and Dmd genes, with the only exception of Myog and Timeless (Tim), which are upregulated genes, with Myog as the most expressed one (*p*-Value = 0.0085; Camerino et al., 2014).

The same trend toward down-regulation is present in the TA muscle obtained at 16-17 weeks of age, when the muscle pathology reaches a stable level; genes with a statistically significant variation are: Arntl2, Ccrn4l, Clock, Egr1, Ror-α, Nr1d2, Csnk1ε, Bhlhe41, Tim, Ppargc1α, and Dmd. Consistently to GC, Myog and Tim showed a statistically significant upregulation, with Myog as the most upregulated gene (*p*-Value = 0.0005, Supplementary Figure 2B). In GC we also observed the loss of mutual variability of expression among Core Clock genes (CCGs).

#### *Mdx* Exercised vs. *mdx* Sedentary

As anticipated before, specific scheduling of exercise could have a damaging effect on the muscle of the mild *mdx* phenotype, leading to a model that better mimics the Duchenne disease. Consequently, a similar comparative analysis was done for exercised *mdx* vs. sedentary *mdx.* Interestingly, an opposite trend of the GC with respect to the TA was found as a main result.

Particularly for GC, exercise deeply changes the signature of all genes compared to untrained *mdx*.

Gastrocnemius muscles show a trend toward upregulation with a statistically significant (*p-*Value < 0.05) variation in: Ccrn4l, Clock, Dbp, Fkbp5, Per2, Per3, Cry1, Ror-α, Nr1d1, Csnk1ε, Csnk1δ, Bhlhe41, Tim, Sirt1, Ppargc1α, and Dmd. Oppositely, the TA muscle gene expression trend shows circadian genes downregulation (Supplementary Figures 2A,B) with statistically significant variation in the following genes: Arntl1, Atf5, Ccrn4l, Dbp, Per3, Cry1, Cry2, Nr1d1, Tim, Sirt1, Ppargc1α.

The different behavior in these two muscles might be explained by the different fiber type composition and different pathology course of the two muscles and their response to the different solicitation of the exercise schedule.

### Gene Prioritization Analysis

**FIGURE 1:**
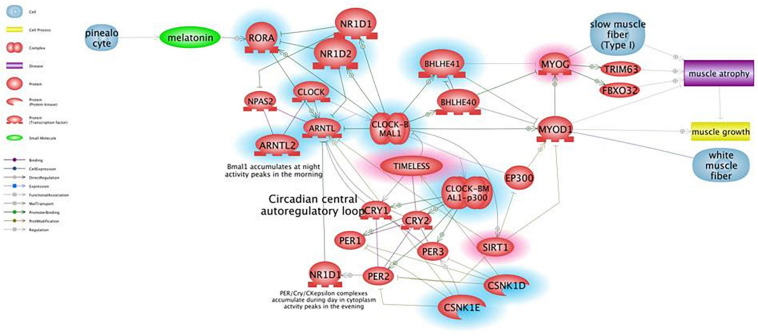
Pathway for circadian rhythm regulation in skeletal muscles and its effect on muscle differentiation and atrophy. Genes that are downregulated in *mdx* mice more than 2-fold are shown in blue, genes upregulated more than 2-fold are shown in red. Pathway Studio analysis shows that downregulation of the ARNTL1 gene leads to upregulation of MYOG, which is known to be involved in muscle atrophy due to increased expression of ubiquitin ligases TRIM63 and FBXO32, rather than being a clear marker of ongoing regeneration. The ARNTL1-CLOCK1 complex is also repressed by upregulation of the Timeless protein that directly binds and represses CLOCK1, and by downregulation of CSNK1E that activates ARNTL1 directly by phosphorylation and through destabilization of PER1/2 proteins.

Figure 1 shows the results of Pathway Studio analysis on circadian rhythm-related genes global deregulation in muscles of *mdx* mice and its effect on muscle differentiation and atrophy. We adopted this tool to build up a gene interactome and compare expression of gene groups between mdx and WT mice in order to prioritized selected genes to be further studied in DMD patients. In the Figure 1 the downregulated genes in *mdx* mice are highlighted with a blue circle while the upregulated gens are highlighted with a red circle; we prioritized only genes with more than 2-fold of deregulation in both groups.

To determine the genes with a statistically significant differential expression (*p*-Value < 0.05), each muscle type (either separately or in pools) was analyzed and the 7 genes that were strongly deregulated in both gastrocnemius and tibialis between *mdx* and WT mice were selected (*Csnk1e, Sirt1, Myog, Myod1, Cry1, Cry2*, and *Arntl*).

### Human Studies

#### Prioritized Circadian Genes: CSNK1E, MYOG, and SIRT1 Are Upregulated in DMD Patients’ Muscle

To evaluate the expression profile of the 7 prioritized genes from mice studies in DMD patients, muscle biopsies from 10 DMD patients with different mutation types and heterogeneous for both age and phenotype severity were selected.

**FIGURE 2:**
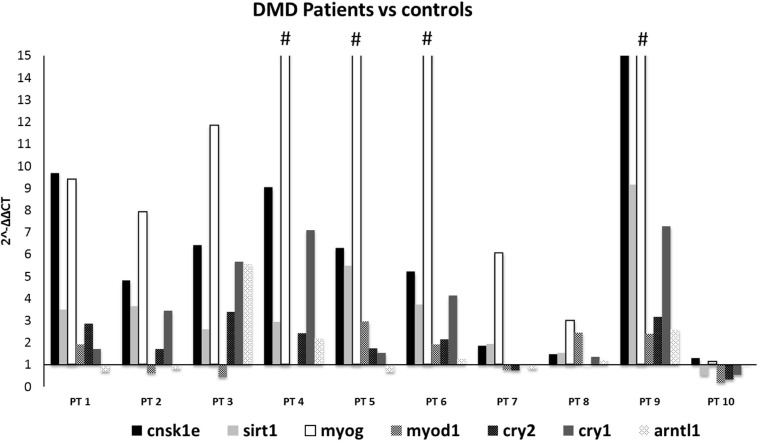
Expression profiling of 7 deregulated genes in muscle samples from DMD patients (*n* = 10). Note the great upregulation of MYOG. (#): indicates that the expression level of MYOG is more than 15-fold, in PT4:25, PT5:34.7, PT6:43, and PT9:34.7. CSNK1E, SIRT1, and MYOG genes were significantly upregulated in all DMD muscles except for PT8 and PT10.

When comparing all DMD muscles with the healthy control, the 7 prioritized genes were deregulated (Figure 2), with levels varying among different samples, possibly due to muscle quality, muscle fiber composition, and disease stage. Specifically, *CSNK1E* (*P*-value = 0.0168), *SIRT1* (*P*-value = 0.0095), and *MYOG* (*P*-value = 0.0072) were significantly upregulated in all DMD muscles, with PT8 and PT10 the only exceptions. The tested genes show a variable level of expression, as expected considering the low number of muscles we studied, however *CSNK1E* and *MYOG* are consistently upregulated in all patients, especially this last one with PT5, PT6, and PT9 reaching levels up to 30 times more with respect to WT. Moreover, a general upregulation of CRY proteins was clearly visible and reflects the downregulation trend seen for ARNTL as part of the negative feedback loop, as already described.

#### CSNK1E Plasma Levels Are Slightly Elevated in Duchenne Patients

Expression data in patients, and in particular upregulation of primarily *MYOG* and then *CSNK1*ε and *SIRT1*, together with the absence of specific studies in Duchenne patients in literature, prompted us to further explore if these deregulated transcripts could reflect in the plasma of patients affected by dystrophinopathies.

For proteomic studies, plasma samples from a total of 16 patients were obtained (14 DMD and 2 BMD), as well as 6 controls were selected and analyzed with CSNK1E -specific ELISA. All DMD patients were ambulant at sampling and homogeneous for age (around 9-10 years on average), except for patient P, who was 19 years old.

Becker muscular dystrophy patients were less homogeneous, as patient A lost ambulation at age 20, while patient B was still ambulant but very young (4 years old).

To draw a standard curve, the mean absorbance of each standard was plotted on the y-axis against the concentration on the x-axis and the best-fit line drawn through the points on the graph.

**FIGURE 3:**
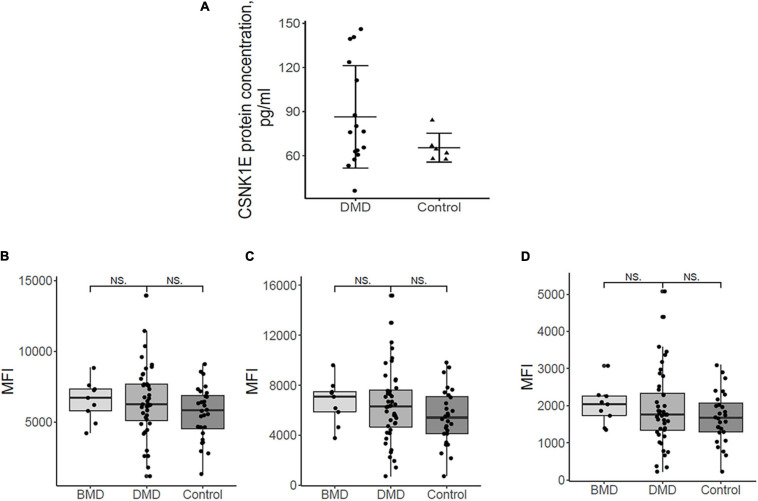
**(A)** Plasma protein dosing of selected upregulated DMD genes. Csnk1ε protein plasma levels in DMD patients (*n* = 16) are slightly elevated with respect to controls (*n* = 6). **(B)** the mean fluorescence intensity (MFI) for sirtuin 1, HPA006295 antibody, and **(C)** MFI for sirtuin 1, HPA007016 antibody. **(D)** MFI for MYOGenin, HPA038093 antibody in DMD, BMD, and controls. Differences between the patient groups are not significant.

CSNK1E protein concentrations of both DMD and controls were depicted in Figure 3A. Figure 3A demonstrated, in general, a slightly elevated plasma level of CSNK1E in all DMD plasma samples with respect to controls.

In order to evaluate a possible relationship between plasma protein levels and other phenotype variables, we checked if phenotype severity (DMD/BMD) mutation types and the age at sampling correlate to CSNK1E protein expression. No meaningful correlations were observed (Supplementary Figure 3).

Nevertheless, three DMD patients (G, H, and I) have about twice the CSNK1E plasma levels, as shown in Supplementary Figure 4A. Notably, higher levels of CSNK1E were seen in two patients with the deletion of exon 45, one of the most frequent mutations localized in one of the well-known mutational hot spots of the dystrophin gene (Supplementary Figure 4B).

To verify if this protein could be a marker of disease severity, plasma concentrations were also evaluated based on age at plasma sampling, but no specific correlation with age, and therefore disease worsening, was shown (Supplementary Figure 4C). However, the sample’s cohort, analyzed for both BMD and DMD patients and healthy controls, proves to be rather limited and an enlargement of the number of the samples could lead to a better definition of a CSNK1E protein profile.

#### SIRT1 and MYOG Plasma Levels Do Not Vary in DMDs/BMDs

Myog and Sirt1 were analyzed in 44 DMD, 9 BMD, and 28 control plasma samples using a suspension bead array assay due to the unavailability of ELISA assays. Figures 3B-D show the abundance of the proteins by mean fluorescence intensity (MFI) for Sirt1, HPA006295 antibody (Figure 3B), and HPA007016 antibody (Figure 3C) and for Myog, HPA038093 antibody (Figure 3D) in DMD, BMD, and controls. Wilcoxon ranked sum test was performed to assess differences in MFI between patient groups. Both proteins were detected in plasma samples but differences between the patient groups were not significant. The large variation of Sirt1 and Myog within patient groups indicates that analyzing a larger cohort would be beneficial. Plasma samples were collected in the morning (8-10 a.m.) and, therefore, we do not expect that a circadian variation might influence the results since release into the blood stream has a circadian pattern.

## Discussion

The *mdx* mouse model of DMD has been widely utilized in evaluating the potential of therapeutic regimens in term of efficacy, efficiency, and side effects. Exploratory biomarkers were extensively searched in the *mdx* mice but also in DMD patients since having available robust biomarkers would greatly benefit the optimization of patient treatments.

Links between circadian rhythms and muscle metabolism are known and rhythmic expression of metabolic factors is common in myogenic homeostatic processes.

Some studies in *mdx* mice are available and pinpoint the role of the circadian clock in muscle differentiation and regeneration. For example, myogenic factors such as *MYOD* and *MYOG* are upregulated in muscle during dark hours and their over expression is suppressed by fasting (Shavlakadze et al., 2013).

Also, MuRF1, Akt1, and ribosomal protein S6 are upregulated in muscles in both fed and fasted mice and for Fbxo32 in fed mice (Chatterjee et al., 2015). Although it is known that skeletal muscle possesses intrinsic functional clocks, it is unclear how it is affected by dystrophinopathy. Asynchrony in fiber regeneration was indeed observed in mdx (Dadgar et al., 2014). Authors found that asynchronously regenerating microenvironments occurred in mice and it may drive fibrosis and regeneration failure. Treatment with either prednisone or Valmorolone (VBP15) mitigated the asynchrony.

Our RNA profiling, using Fluid-CIRC containing 32 genes related to circadian rhythms and muscle regeneration, showed that the majority of the explored circadian genes were profoundly downregulated in sedentary animals with a similar trend in the gastrocnemius and tibialis muscles.

We also observed the upregulation of *Tim* in both TA and GC, and we hypothesize that this might occur via *Per1* and *Per2* deep downregulation, as seen in the Fluid-CIRC. Indeed, Tim is part of the negative loop of inhibition of Clock/Arntl1-activated PER proteins.

Tibialis anterior and GC in untrained mice behave similarly. We showed that both GC and TA lack the physiological mutual expression variability among Core Clock Genes (CCGs), which is due to the network regulation based on feedback loops. It is true that different ages of TA and GC muscles may reflect different stages of disease (early in GC and later in TA), nevertheless, gene expression tendency is similar.

Underlining muscle-specific differences, circadian genes were highly upregulated in gastrocnemius but mainly downregulated in tibialis in exercised mice. The very different fiber composition of these two muscles may also account for this opposite sign of expression in trained animals (Schiaffino and Reggiani, 2011; Bloemberg and Quadrilatero, 2012). Considering the consistent pattern between TA and GC that we observed in untrained *mdx* and the deregulation of their expression in the exercised GC muscle, we prioritized 7 genes as the most profoundly affected: *CSNK1E, SIRT1, MYOG, MYOD, CRY1, CRY2*, and *ARNTL1* (Bmal). Realtime assay in 10 DMD patient muscle biopsies showed that all of these genes were persistently upregulated and have tight interactions within the circadian pathway, which is shown in Figure 2. SNEA enrichment analysis underlines a major role of genes involved in muscle mass maintenance. This function is exerted through the central circadian feedback loop (Per and Cry circuit under Clock and Bmal oscillators), which influences muscle growth mainly on fast type fibers (FIGURE muscle) via *MYOD* positive regulation. Slow fibers are mainly under the influence of *MYOG*, which is directly and positively regulated by *MYOD*, but also indirectly interacts with Clock/Bmal oscillators (Aoyama and Shibata, 2017). The upregulation of *MYOG* may also contribute to causing muscle atrophy due to increased expression of ubiquitin ligases *TRIM63* and *FBXO32* (Moresi et al., 2010; Macpherson et al., 2011). Additional repression of ARNTL1-CLOCK1 complex is achieved by upregulation of Tim protein that directly binds and represses *CLOCK1*, and by downregulation of *CSNK1E* that activates *ARNTL1* directly by phosphorylation and through destabilization of PER1/2 proteins. The master clock transcription activator, *ARNT*-like 1 (*BMAL1*), which shows a clear upregulation trend in the muscle of both exercised *mdx* mice and patients, has a very important role in muscle, not only by participating in the maintenance of muscle mass but also in fiber regeneration. Indeed, genetic ablation of *BMAL1* in engineered injured muscle significantly impairs fiber regeneration with markedly suppressed new myofiber formation and attenuated myogenic induction (Chatterjee et al., 2015) and, specifically in *mdx* mice, it was demonstrated that the loss of Bmal1 aggravates the disease phenotype by increasing creatin kinase levels and injury areas and decreasing muscular force (Hongbo et al., 2020).

A lower satellite cell number (regeneration block) was observed in Bmal1-null mice but also in skin (Plikus et al., 2015), cardiomyocytes (Durgan and Young, 2010), and hepatocytes (Yan et al., 2010). The observed upregulation of *MYOD* and *MYOG* in DMD muscles is interesting and predicts to force up muscle differentiation and regeneration (Ji et al., 2009; Cappellari et al., 2020). *MYOG* is an extremely well-known transcription factor that modulates muscle regeneration. Together with *MYF5*, *MYOD*, and *MRF*, *MYOG* promotes specification of the muscle satellite cell lineage. These cells are vital for muscle since they become the resident stem cell compartment in adult tissue and, therefore, the source of regeneration. It is conceivable that severe damage in DMD muscle activates a compensating upregulation of *MYOD* and *MYOG* in order to allow more muscle fiber replacement. However, while *MYOG* upregulation may result in the activation of satellite cells, it may also be detrimental to muscle since forcing myogenesis may inhibit the satellite cells’ self-renewing process (Almada and Wagers, 2016). Also, *SIRT1* and *CSNK1E* were upregulated in DMD muscles. *SIRT1* is a histone deacetylase acting on stress resistance and cell survival. In *mdx*, *Sirt1* activation induces a series of positive effects such as reduction of both oxidative stress and inflammation, fast to slow myofiber switching and less fibrosis. *Sirt1* is under direct control of Clock/Bmal and its expression/activation may also represent a compensatory response of myofiber to ongoing oxidative stress and inflammation. Nothing is known about Csnk1ε protein and skeletal muscle interaction; it positively influences *Per* and *Cry* genes and is thought to negatively affect the transcription-translation-based clock auto-regulatory loop (Liu et al., 2016).

Observing the RNA profile in untrained *mdx*, the 7 selected genes are downregulated. Similarly to DMD, these genes are also involved in muscle differentiation and regeneration in mice (Almada and Wagers, 2016). The opposite sign of expression in humans and sedentary mice might be linked to the nature of myofiber in *mdx* and DMD, as well as to the (partial) preservation of satellite fibers in *mdx*, although in an age-dependent manner. The protocol of forced treadmill exercise moved the expression profile of mdx GC muscle toward that observed in DMD patients, supporting that the failure in mechanical-metabolic signaling and function is a hallmark of dystrophic severity (Camerino et al., 2014; Capogrosso et al., 2016) and is also related to deregulation in clock gene expression.

Based on the data we obtained in DMD patients’ muscle, we evaluated if RNA deregulation might also be mirrored in protein from plasma samples. We showed that CSNK1E, MYOG, and SIRT1 are all detectable in human (control and DMD) plasma but only CSNK1E abundance differs slightly between DMD patients and healthy individuals, especially for DMDs carrying deletions involving exon 45. Although the concentration of CSNK1E varies within both the patient group and the control group, these findings suggest that CSNK1E might be an exploratory circadian biomarker for DMD, measurable in plasma. Circadian gene products such as blood biomarkers and their diurnal fluctuations have never been described and remain deserving of further investigation.

## Conclusion

In conclusion, our study underlines upregulation of *CSNK1E*, *SIRT1*, and *MYOG* in both mdx and DMD skeletal muscle, suggesting that alterations of circadian circuits may activate a more severe and dystrophic-like muscle pathology. We also have shown that CSNK1E, MYOG, and SIRT1 circadian proteins are measurable in plasma. Circadian genes deserve to be further studied as disease-related readout parameters linked to disease status or disease severity. Due to their tight link to muscle differentiation and regeneration, they may also be relevant drug targets in DMD.

## Data Availability Statement

The authors acknowledge that the data presented in this study must be deposited and made publicly available in an acceptable repository, prior to publication. Frontiers cannot accept a manuscript that does not adhere to our open data policies.

## Ethics Statement

The studies involving human participants were reviewed and approved by the S. Anna University Hospital of Ferrara Ethics Committee (no. 02/2009, 26th February 2009). Written informed consent to participate in this study was provided by the participants’ legal guardian/next of kin. The animal study was reviewed and approved by Department of Pharmacy-Drug Sciences, University of Bari (Italy).

## Author Contributions

HO and CS performed the mice experiments. HO, RR, and MF performed the human muscle experiments. PM and AD performed the mice *in vivo* studies. ES and AY performed the bioinformatics analysis and gene prioritization. EM, EB, AA, and AD’A performed the patient clinical studies. MF, RR, CJ, and CA-K performed the immunoassay studies. MM provided the muscle samples from the Telethon Biobank. RR, AD, and AF wrote the manuscript. AF conceived and supervised the work. All authors contributed to the article and approved the submitted version.

## Conflict of Interest

ES was employed by company Ami-Go-Science LLC. AY was employed by company Elsevier. The remaining authors declare that the research was conducted in the absence of any commercial or financial relationships that could be construed as a potential conflict of interest.

## Footnotes

1 www.dmd.nl

2 https://www.graphpad.com/quickcalcs/ttest1/

3 www.treat-nmd.eu

## Supplementary Material

Supplementary Figure 1 Design of the TaqMan Low Density Array (TLDA) micro fluidic-card (Fluid-CIRC). The Fluid-CIRC is a 384-count well plate pre-loaded with TaqMan gene expression assays of 32 murine genes involved in circadian rhythm, muscle regeneration, metabolism, apoptosis, immune reaction, and cellular proliferation, plus ACTIN-B and GAPDH as reference genes.

Supplementary Figure 2 Fluid-CIRC expression data of Gastrocnemius GC **(A)**, and Tibialis anterior TA **(B)** of WT (blue bars), *mdx* sedentary (red bars) and *mdx* exercised (green bars) samples. Mean standard error (SEM) is indicated with error bars. In *mdx* CCGs are deregulated with a trend through a general downregulation. Genes with a statistically significant variation (*p*-Value < 0.05) are indicated with asterisk (^∗^). Only MYOG is consistently upregulated in both muscle types. Comparing mdx-sedentary vs. mdx-exercised, we can observe an opposite behavior of the two selected muscle types. MYOG and MYOD don’t show a consistent change of expression with respect to *mdx.*

Supplementary Figure 3 Csnk1ε protein plasma levels in DMD patients analyzed with respect to phenotype **(A)**, mutation type **(B)**, and age **(C)**. White bars are averaged controls (protein concentrations measured in the 6 controls were averaged and used for the comparison), gray bars are BMD patients (*n* = 2) and black bars are DMD patients (*n* = 14).
