# Supplementary figures and images for: Circadian Genes as Exploratory Biomarkers in DMD: Results From Both the mdx Mouse Model and Patients
Source: Front Physiol. 2021 Jul 8;12:678974. doi: 10.3389/fphys.2021.678974 (PMC8300012; doi:10.3389/fphys.2021.678974)

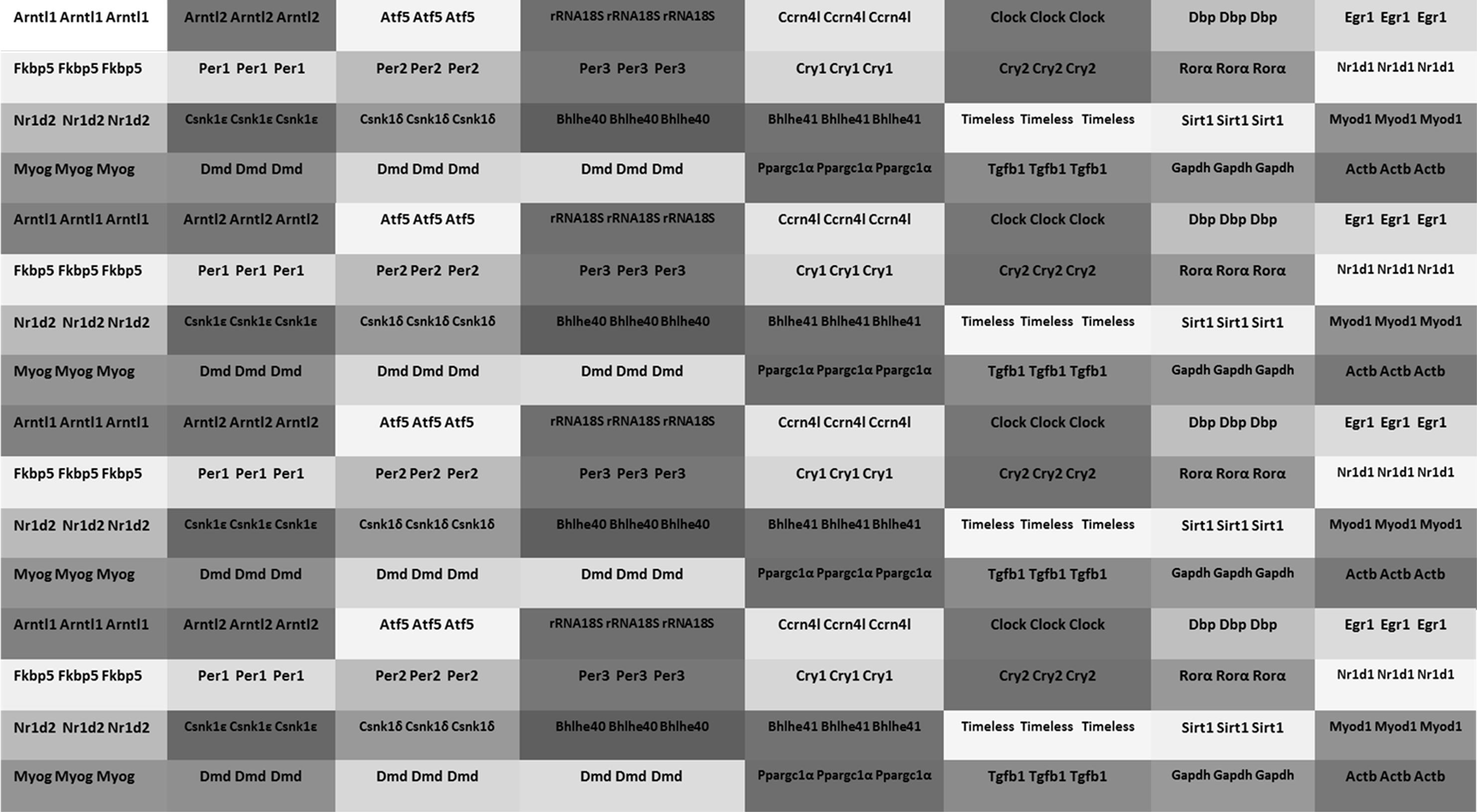

Supplement: Supplementary Figure 1 — Design of the TaqMan Low Density Array (TLDA) micro fluidic-card (Fluid-CIRC). The Fluid-CIRC is a 384-count well plate pre-loaded with TaqMan gene expression assays of 32 murine genes involved in circadian rhythm, muscle regeneration, metabolism, apoptosis, immune reaction, and cellular proliferation, plus ACTIN-B and GAPDH as reference genes. [file Image_1.TIF]

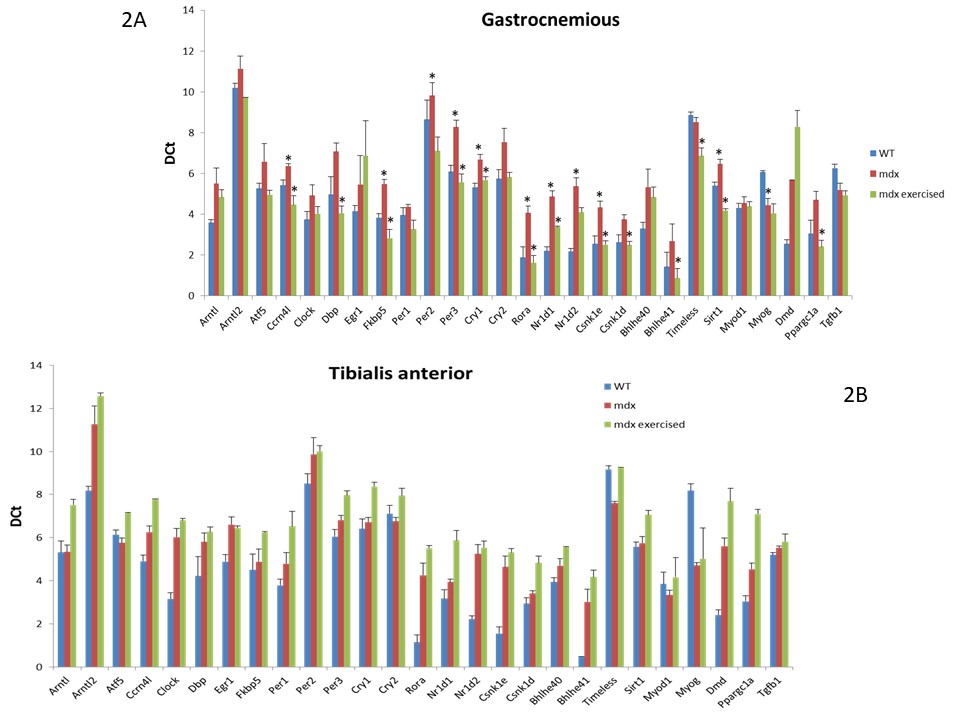

Supplement: Supplementary Figure 2 — Fluid-CIRC expression data of Gastrocnemius GC (A), and Tibialis anterior TA (B) of WT (blue bars), mdx sedentary (red bars) and mdx exercised (green bars) samples. Mean standard error (SEM) is indicated with error bars. In mdx CCGs are deregulated with a trend through a general downregulation. Genes with a statistically significant variation (p-Value < 0.05) are indicated with asterisk (∗). Only MYOG is consistently upregulated in both muscle types. Comparing mdx-sedentary vs. mdx-exercised, we can observe an opposite behavior of the two selected muscle types. MYOG and MYOD don’t show a consistent change of expression with respect to mdx. [file Image_2.JPEG]

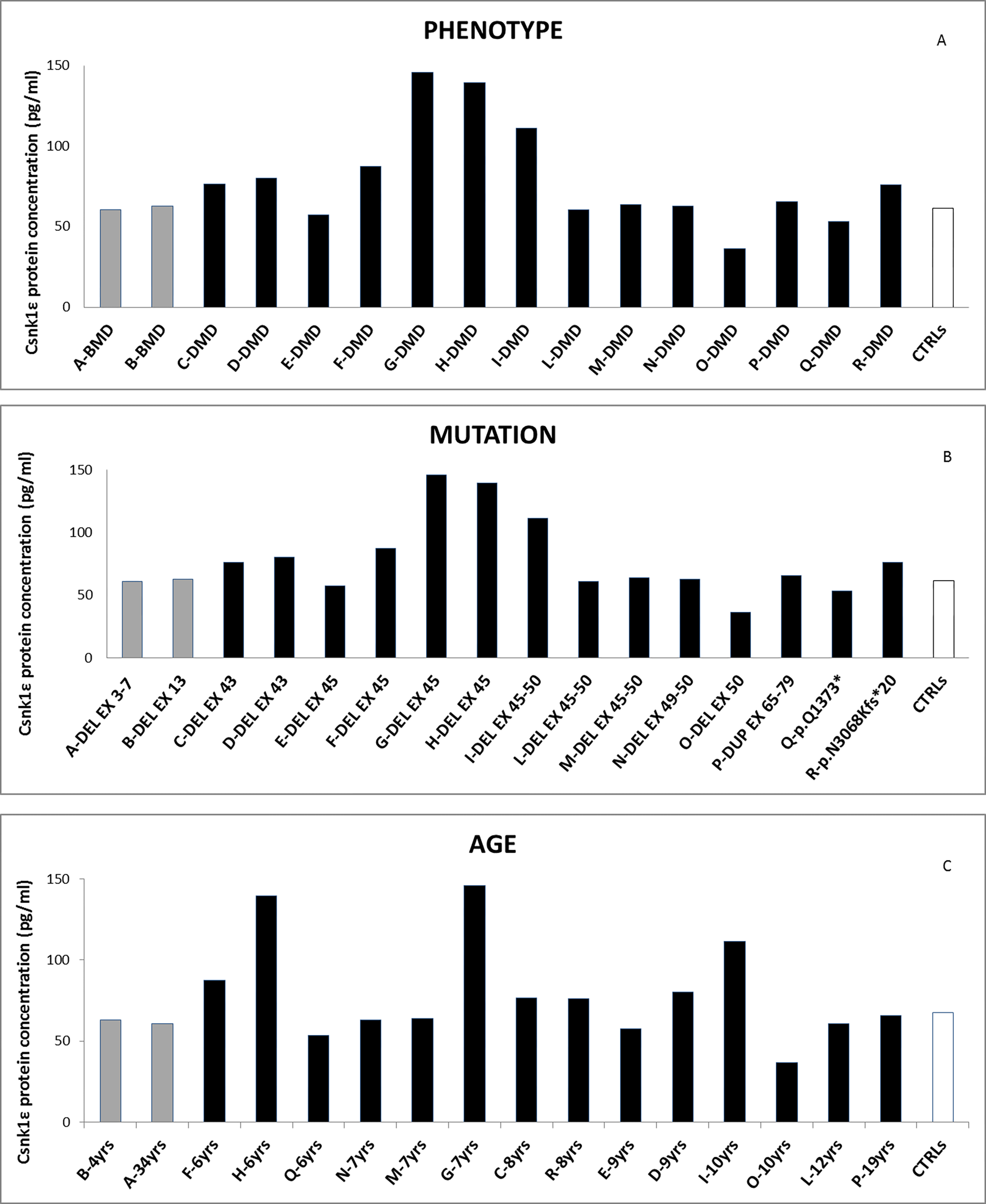

Supplement: Supplementary Figure 3 — Csnk1ε protein plasma levels in DMD patients analyzed with respect to phenotype (A), mutation type (B), and age (C). White bars are averaged controls (protein concentrations measured in the 6 controls were averaged and used for the comparison), gray bars are BMD patients (n = 2) and black bars are DMD patients (n = 14). [file Image_3.TIF]
